# Effect of slurry composition on the chemical mechanical polishing of thin diamond films

**DOI:** 10.1080/14686996.2017.1366815

**Published:** 2017-09-15

**Authors:** Jessica M. Werrell, Soumen Mandal, Evan L. H. Thomas, Emmanuel B. Brousseau, Ryan Lewis, Paola Borri, Philip R. Davies, Oliver A. Williams

**Affiliations:** ^a^ School of Physics and Astronomy, Cardiff University, Cardiff, UK.; ^b^ Cardiff School of Engineering, Cardiff University, Cardiff, UK.; ^c^ School of Biosciences, Cardiff University, Cardiff, UK.; ^d^ School of Chemistry, Cardiff University, Cardiff, UK.

**Keywords:** Chemical mechanical polishing, nanocrystalline diamond, surface roughness, chemical vapour deposition

## Abstract

Nanocrystalline diamond (NCD) thin films grown by chemical vapour deposition have an intrinsic surface roughness, which hinders the development and performance of the films’ various applications. Traditional methods of diamond polishing are not effective on NCD thin films. Films either shatter due to the combination of wafer bow and high mechanical pressures or produce uneven surfaces, which has led to the adaptation of the chemical mechanical polishing (CMP) technique for NCD films. This process is poorly understood and in need of optimisation. To compare the effect of slurry composition and pH upon polishing rates, a series of NCD thin films have been polished for three hours using a Logitech Ltd. Tribo CMP System in conjunction with a polyester/polyurethane polishing cloth and six different slurries. The reduction in surface roughness was measured hourly using an atomic force microscope. The final surface chemistry was examined using X-ray photoelectron spectroscopy and a scanning electron microscope. It was found that of all the various properties of the slurries, including pH and composition, the particle size was the determining factor for the polishing rate. The smaller particles polishing at a greater rate than the larger ones.

## Graphical abstract

**Figure:**
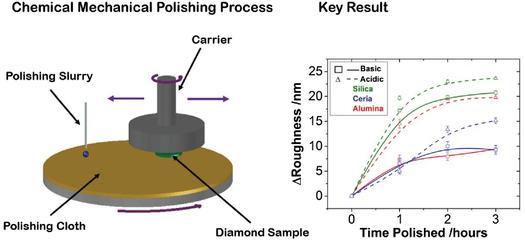

## 1. Introduction

Nanocrystalline diamond (NCD) thin films grown by chemical vapour deposition (CVD) are able to retain many of the properties of single crystal diamond and can be produced with high quality at relatively low cost [1]. These two properties have generated an interest for using NCD thin films in a number of areas such as tribology, optical coatings, electrochemistry, thermal management, surface acoustic wave (SAW) devices and micro-electro-mechanical systems (MEMS) [2]. To align with current fabrication techniques, NCD thin films are grown on non-diamond substrates such as silicon. Successful and efficient growth on the majority of foreign substrates requires a nucleation enhancement step. One such method involves seeding the substrates with detonation nanodiamond (DND) particles [3]. During CVD growth these DND particles then grow via a Volmer–Weber model [4], i.e. the DND particles grow longitudinally and laterally in the plane on the surface of the substrate until they coalesce to form a film. This film will comprise diamond grains of varying sizes and facet orientations. CVD growth for each grain from this point onwards will be predominantly normal to the surface. The growth rate of diamond grains will depend upon their facet orientation in accordance with the Van der Drift growth model [5]. As has alreadybeen stated, there is a variation in facet orientation between each individual diamond grain. As a result there is a variation in growth rate between the different grains. This leads to competitive growth between the individual grains and results in an uneven final film layer, or surface roughness, which evolves with film thickness. This surface roughness acts as a significant barrier to the construction and performance of NCD thin films for various applications, such as SAW devices and MEMS to name but two [6].

One method to reduce this surface roughness involves interrupting the crystal growth and limiting the maximum size of the diamond grains. This is done by reducing the 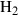 and/or increasing the 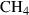 content of the plasma. This also leads to an increase in the 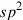 content of the material at the grain boundaries which in turn reduces the Young’s modulus [1,7,8]. Also for films grown under these conditions it is difficult to achieve a root mean square (RMS) roughness lower than 5 nm [2]. This figure still exceeds the accepted limit for the fabrication for their various applications [6,9] and so restricts the use of these films.

Another technique involves etching away the silicon substrate and using the nucleation diamond grains as the surface [10]. The surface roughness will be significantly less this side of the diamond film than the other. Unfortunately the quality of this side of the diamond film is inferior to the upper coalesced film with reduced values of Young’s modulus and thermal conductivity [10]. This technique also requires thick free standing films and complex fabrication processes [10].

Alternatively, surface roughness can be reduced through polishing the as-grown rough NCD thin films. Historically, diamond polishing has involved the use of diamond on diamond in a contact mechanical polishing mechanism [11]. This process is impractical for NCD thin films because it produces uneven wear rates and the films are susceptible to shattering. The uneven wear rate is a result of the mechanical polishing of diamond being highly dependent upon both the crystallographic orientation of the diamond and the azimuthal angle of polishing [12,13]. As stated, the surface roughness of NCD thin films is a result of crystallographic orientation variation; therefore, this type of polishing can lead to protruding crystals left behind on the film surface [14]. Potential shattering is the result of the high pressures typical of mechanical polishing and the characteristic wafer bow of NCD thin films [11]. There is an initial wafer bow always present in the substrate which is then compounded during cooling after CVD growth as a result of the difference in the coefficient of expansion between the diamond film and its non-diamond substrate [15,16]. The bow then places additional stress on the NCD thin film, especially when pushed against planar surfaces, making it more susceptible to damage whilst being subjected to heavy mechanical polishing.

To overcome these issues, the gentle chemical mechanical polishing (CMP) technique was adapted by Thomas et al. [17]. This technique utilises a soft polyester based pad as the polishing base and a silica based colloid as the polishing slurry at room temperature. It is a commonly used technique in the integrated circuit (IC) fabrication industry for polishing silicon wafers [18] and has been shown to polish both NCD [17] and bulk single crystal diamond (SCD) successfully [19].

In both of the aforementioned studies, a basic polishing slurry containing silica (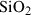) particles was used. In the present study, three different polishing particles, ceria (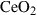), alumina (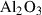) and silica, all common to the IC fabrication polishing industry, were used to examine how composition affects the polishing rates. For each particle type there was an acidic (pH 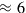) and a basic (pH 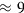) slurry. The aim of this study was to compare and contrast the different slurries to understand better how silica, a material with a density of 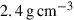 and a hardness of 7 [20] on the Mohs scale, is able to polish diamond of density 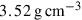 [11] and hardness 10 [20]. The significant difference in hardness and density does not make the polishing far-fetched, considering that harder materials can be polished by softer particles [21–23], e.g. tantalum by silica [23].

## 2. Experimental procedure

For this study, a series of 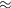
360 nm thick NCD thin films were grown on a 500 nm thick buffer layer of silicon dioxide that coated a 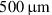 thick p-type silicon (100) wafer 2-inches in diameter. Before growth, these wafers were cleaned using the standard SC-1 process [24] of 30% 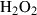 : 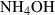 : deionised (DI) 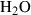 (1:1:5) at 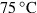 for 10 minutes. The substrates were then rinsed in DI 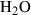 in an ultrasonic bath for 10 minutes and spun dry. For the seeding step, the wafers were placed in a mono-dispersed nanodiamond (with a diameter of 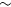
5 nm) and DI 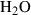 colloid which was then agitated in an ultrasonic bath for 10 minutes. This processes encourages the nanodiamond particles to bond to the surface of the substrate via electrostatic attraction and is known to produce nucleation densities exceeding 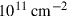 [3]. After this the wafers were rinsed, spun dry at 3000 rpm, and then immediately placed inside the CVD chamber. CVD was carried out in a Seki 6500 series microwave plasma reactor under 3% 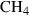/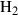 conditions at 47 Torr and 4.2 kW microwave power. Upon termination of growth, the films were cooled down in hydrogen plasma to ensure hydrogen termination and prevent deposition of non-sp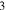 material. Substrate temperatures were 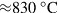 as determined by a dual-wavelength pyrometer, with substrate heating solely from the microwave induced plasma. Film thickness was determined *in situ* through the use of laserinterferometry, and *ex situ* with a Filmetrics F-20 Spectral Reflectance system. The system was modelled as roughness on diamond on silicon dioxide on silicon. Known values of the wave number *k* and refractive index *n* were used for the diamond, silicon dioxide and silicon from the Filmetrics database to determine the thickness. The RMS roughness of each film was measured using a Park Systems Park XE-100 atomic force microscope (AFM) with a Tespa-V2 tip in non-contact mode. The *XY* spatial resolution was 20 nm and the *Z* spatial resolution was 0.2 nm. The AFM images were analysed using the Park Systems software programme XEI Image Processing. An average of three 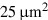-pagination areas of the as-grown films showed that they all had an approximate RMS average of 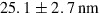.

Three different polishing particles were used: ceria (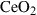), alumina (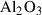) and silica (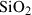). For each particle type there was an acidic (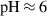) and a basic (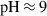) slurry. The basic silica polishing slurry was SF1 Polishing Fluid from Logitech Ltd., which was made acidic in house through the addition of phosphoric acid (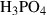). The alumina particle based polishing slurries were supplied by Saint Gobain Ltd., brand names Polycrystalline Alumina Polishing Slurries 9240 and 9245. Finally, the ceria based polishing slurry was supplied by Eminess, brand name Ultra-Sol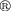 Optiq, which arrived basic and was also acidified. Table 1 shows a summary of the specific properties of each individual slurry. The particle size and particle content was provided by the slurry manufacturers. The particle diameters were also measured using a dynamic light scattering (DLS) technique. To do this the slurries were diluted to a 1/1000 ratio and then measured using a Malvern Instruments Ltd. Zetasizer Nano Z device. pH values were measured using a Mettler Toledo™  FG2 FiveGo™  Portable pH Meter.

**Table 1.** Properties of slurries. Density and hardness values taken from [20]. The particle size and particle content were provided by the slurry manufacturers. DLS particle diameters and pH were measured in this work.

Property	Silica	Alumina	Ceria
Density ()	2.2–2.6	4.0	7.1
Hardness (Mohs)	6–7	9	6
260mmParticle diameter measured using DLS ()	0.1 (basic)	1.0 (basic)	0.5 (basic)
	0.1 (acidic)	0.2 (acidic)	0.5 (acidic)
260mmParticle size according to manufacturer ()	2*Unavailable	0.4 (basic)	0.4 (basic)
		0.3 (acidic)	0.4 (acidic)
2*Particle content ()	2*15–50	20 (basic)	20 (basic)
		20 (acidic)	20 (acidic)
260mmpH of solution	9.6	9.1	8.9
	5.6	5.8	5.8

The NCD films were polished by CMP using a Logitech Ltd. Tribo CMP System in conjunction with a SUBA™ X polishing pad and the chosen slurry, at intervals of one hour for a total of three hours. Before use, the polyester polishing pad was conditioned for 30 minutes using an abrasive conditioning chuck consisting of a nickel plate embedded with diamond grit, and DI 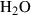 to ensure it had a high surface roughness for maximum polishing action and slurry distribution [25]. During film polishing, both the pad and carrier were kept at 60 rpm rotating in opposite directions, while the carrier swept across the pad. Down pressure was kept at 2 psi, while a backing pressure of 20 psi was applied in order to account crudely for the NCD film bow. After initial wetting of the plate, the feed slurry rate was kept at 40 ml/min. At hourly intervals the films underwent a clean using the standard 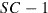 process and their roughness and thickness were measured.

For comparison of the surface chemistry of each film after the maximum duration of polishing, X-ray photoelectron spectroscopy (XPS) data was taken using a Thermo Scientific™K-Alpha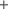 spectrometer. Spectra were acquired using a monochromatic Al source operated at 72 W (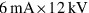) and at pass energies of 40 and 150 eV for high resolution and survey scans, respectively, over an analysis area of 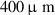. Charge compensation was achieved using the K-Alpha charge neutralisation system, which employs a combination of both electrons and low energy argon ions. Spectra requiring charge neutralisation were subsequently calibrated to the C1s line at 285.0 eV. The results were analysed using the software CasaXPS. Scanning electron microscope (SEM) images were also taken of the final films using the in-lens detector of a Raith eLine system operated at 10 kV and a working distance of 10 mm.

**Figure 1.**
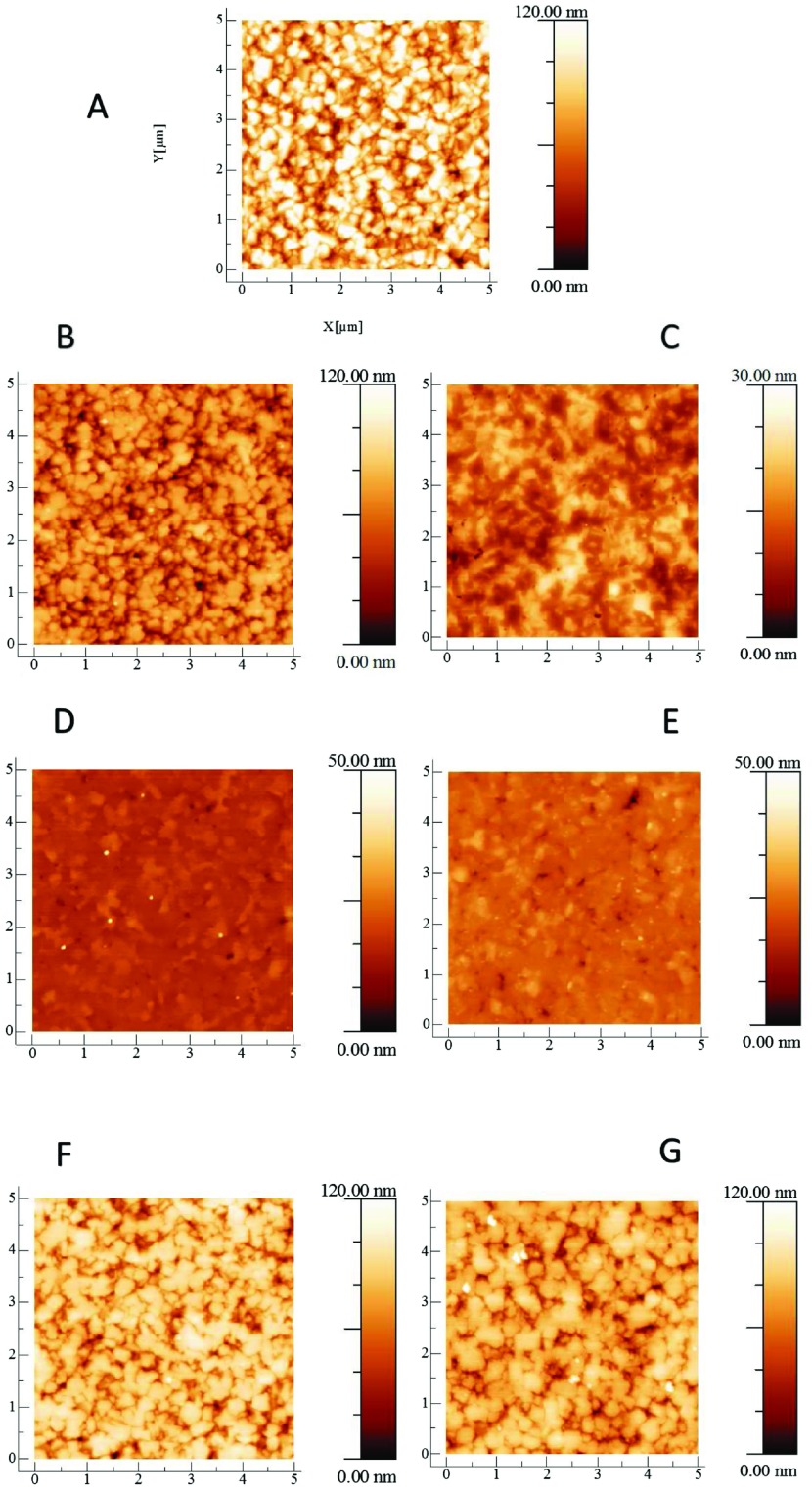
AFM images of the NCD films. (A) Shows the as-grown NCD film and is included here for comparison with the polished films. (B) NCD film after three hours of basic alumina polishing. (C) NCD film after three hours of acidic alumina polishing. (D) NCD film after three hours of basic silica polishing. The small white dots on the surface of Figure 1(D) are due to dust particles as a result of the samples exposure to air. They are only visible because the diamond surface is so smooth that minor particles can be observed as perturbations. They are few in number and sufficiently minor that they are unlikely to affect the AFM measurement significantly. (E) NCD film after three hours of acidic silica polishing. (F) NCD film after three hours of basic ceria polishing. (G) NCD film after three hours of acidic ceria polishing. There is a strong variation in the roughness reduction of each film for the different slurries. The films polished by basic and acidic silica – (D) and (E), respectively – are significantly smoother than the as-grown film (A). Whereas the surface polished by basic ceria (F) and the surface polished by acidic ceria (G) look similar to the as-grown film. Interestingly, the film polished by basic alumina looks similar to the surfaces polished by ceria – (G) and (F) – whilst the film polished by acidic alumina (C) looks smoother than these but not as smooth as the silica-polished surfaces – (D) and (E).

**Figure 2.**
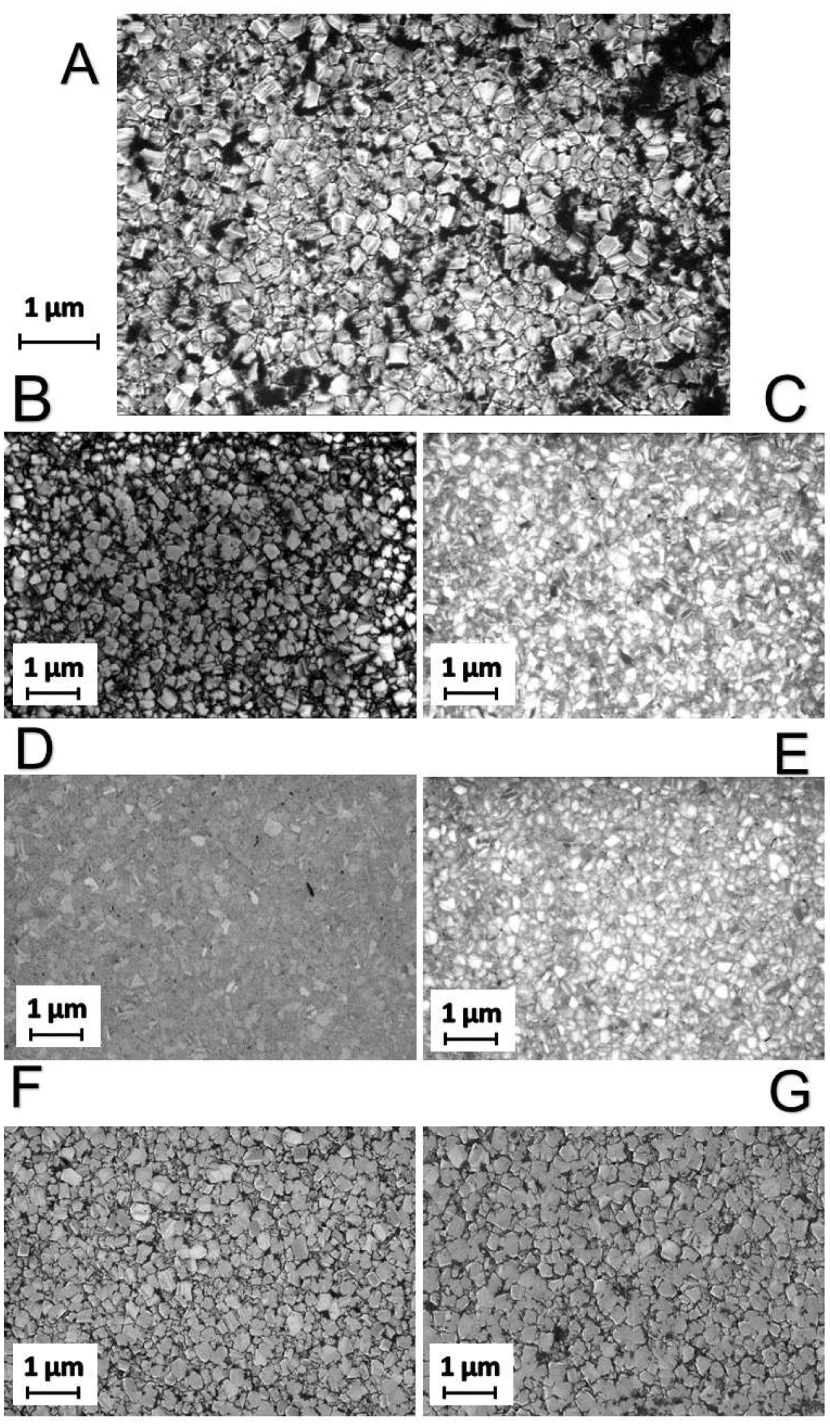
SEM images of the NCD films. (A) Shows the as-grown NCD film and is included here for comparison with the polished films. (B) NCD film after three hours of basic alumina polishing. (C) NCD film after three hours of acidic alumina polishing. (D) NCD film after three hours of basic silica polishing. (E) NCD film after three hours of acidic silica polishing. (F) NCD film after three hours of basic ceria polishing. (G) NCD film after three hours of acidic ceria polishing. It is clear just from these images that there is a variation in the roughness reduction of the different slurries. The films polished by basic silica (D), acidic silica (E) and acidic alumina (C) are significantly smoother than the as-grown film (A). Whereas the surfaces polished by basic ceria (F), acidic ceria (G) and basic alumina (B) look similar to the as-grown film. Interestingly, there is a variation between the acidic and basic slurries containing alumina particles but not for the other two particles. These results agree with the AFM measurements.

## 3. Results and discussion

### 3.1. Morphology

Figure 1 shows AFM images of seven NCD thin films and Figure 2 shows SEM images of the same set of films. Image (A) for each case is the as-grown film and is included in the set for comparison against six polished films. As stated in the caption to Figure 1, the small white dots on the surface of Figure 1(D) are dust particles; they are only visible because the surface is so smooth that minor particles can be observed as perturbations. Rougher AFMs do not show these dots due to tip convolution and the dust particles’ being very small compared to surface roughness. The dust particles are few in number and sufficiently minor that they are unlikely to affect the AFM measurement significantly. If there were any other type of defect on the surface they would have appeared on the SEM image of the same sample but, as can be seen from Figure 2(D), they do not. It is clear just from these images that there is a significant variation in the roughness reduction of the different slurries. The surface of the NCD thin film polished by basic silica (D) and the surface of the NCD thin film polished by acidic silica (E) are significantly smoother than the as-grown film (A). Whereas the surface polished by basic ceria (F) and the surface polished by acidic ceria (G) look similar to the as-grown film. Interestingly the surface polished by basic alumina looks similar to the surfaces polished by ceria – (G) and (F) – whilst the surface polished by acidic alumina (C) looks smoother than these but not as smooth as the silica-polished surfaces (D) and (E). This can be seen from both the AFM and the SEM images.

**Figure 3.**
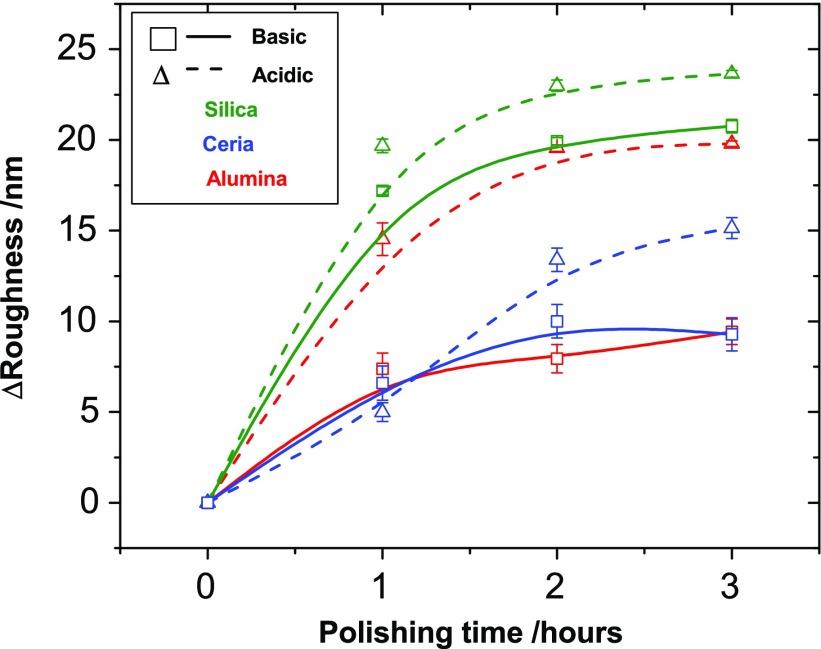
The reduction in the RMS roughness of each film after each hour of polishing up to a total of three hours. The average RMS of three 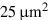 areas of each polished film is plotted with their respective standard deviations, the zero mark being the roughness of the as-grown diamond film. It can be seen clearly that, regardless of particle type, the acidic slurry always leads to a greater RMS roughness reduction compared to its basic counterpart. However, the variation between basic and acidic is inconsistent between particles, suggesting that pH alone cannot be a deciding polishing factor. The slurries containing silica particles polish at greater rates than the ceria particles but there is an inconsistency here with the alumina slurries. This large variation between the alumina slurries means particle composition is not the only deciding polishing factor like pH.

**Figure 4.**
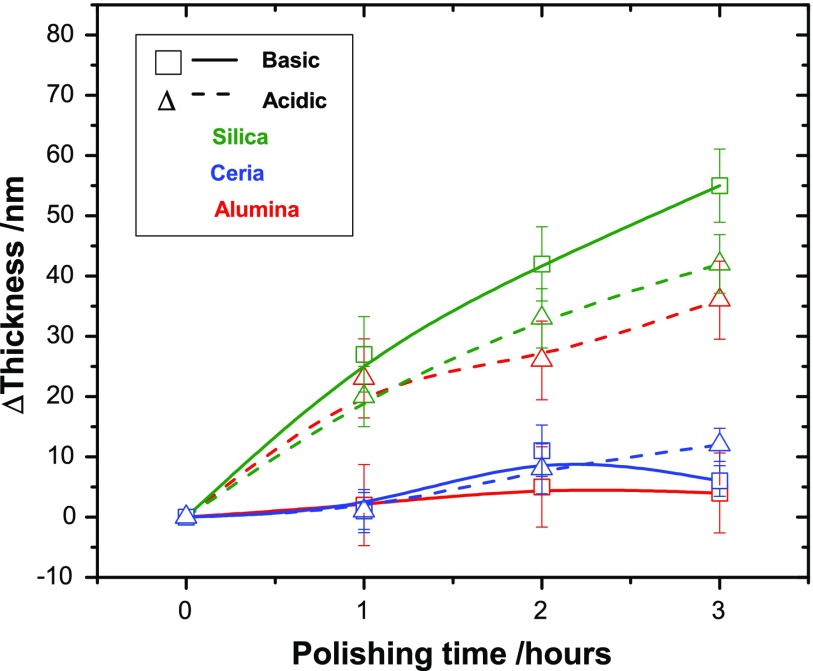
The change in the thickness of the NCD films during the three hours of polishing, the zero mark being the thickness of the as-grown diamond film. For those slurries that polished with the greater rate (see Figure 3) there is also a corresponding greater reduction in the thickness of the diamond. This is to be expected as the polishing removes the diamond peaks.

Figure 3 graphically shows the reduction in the RMS roughness of each film after each hour of polishing up to a total of three hours. The average RMS of three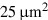 areas of each polished film is plotted with their respective standard deviations. Figure 4 shows the corresponding thickness reduction. For those slurries that polished with the greater rate there is also a corresponding greater reduction in the thickness of the diamond. This is to be expected as the polishing is removing the roughness layer in order to produce a more uniform film. The zero mark for each graph being the roughness and thickness respectively of the as-grown diamond film. Only three hours of polishing were performed for this study because wear rate was seen to drop significantly after the first hour and an observable difference in polishing was recorded within this time period.

From Figure 3, it is clear that the acidic slurries polish at a greater rate than their basic counterparts; however, the degree to which they polish varies widely. In the three hours, the acidic silica polishing slurry reduced the surface roughness by 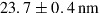, the basic version of this slurry reduced surface roughness in the same time by 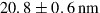. Whilst not within errors this is at most a difference of 3.8 nm. In contrast, the alumina particles show a difference of 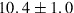 nm. The ceria particles show 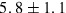 nm. The variation is not consistent between the particles, showing that the pH alone cannot be a deciding polishing factor.

A comparison of the results considering only the particle composition shows that the silica particles polish at greater rates than the ceria particles but there is an inconsistency here with the alumina slurries. The acidic alumina polishes at a rate similar to the silica but the basic alumina at a rate comparable to ceria. This large variation between the alumina slurries means particle composition, and therefore their particles properties such as Mohs hardness and density, is not the only deciding polishing factor.

**Figure 5.**
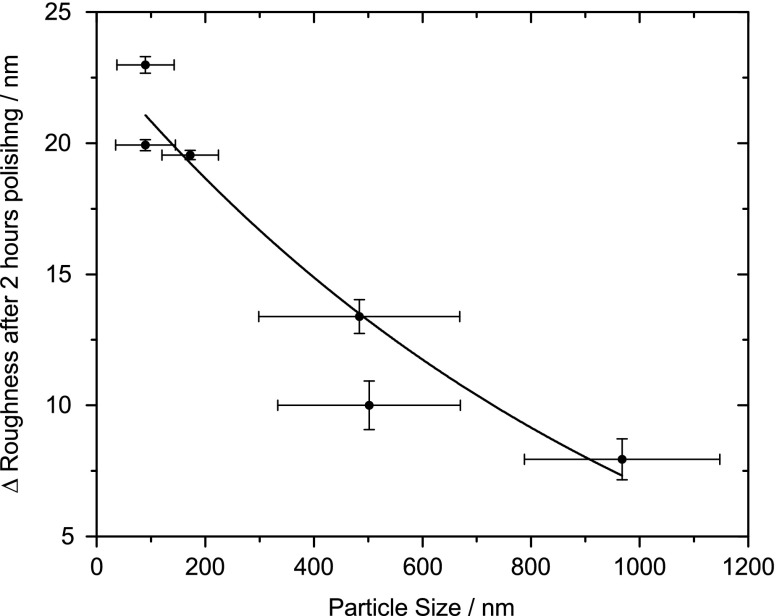
Change in the RMS roughness after two hours polishing compared against the size of the polishing particles and their respective standard deviation, measured by DLS. There is a clear correlation between particle size and rate. Smaller particles, with a diameter less than 200 nm, polish NCD thin films at a greater rate than the larger ones with a diameter greater than 500 nm.

Comparing Figure 3 with the other known slurry properties in Table 1, a relationship between polishing rate and particle size is revealed. Figure 5 plots the diameter of the respective particles and their standard deviation, as measured by DLS, against the change in roughness after two hours of polishing. There is a clear correlation between the diameter and rate, with the smaller particles polishing faster than the larger particles. The faster rate is most likely due to an increase in contact area as a result of the use of smaller sized particles. The percent particle contents of the slurries are within an order of magnitude of each other, which means that those slurries with smaller particles will also contain a greater number of the said particles.

### 3.2. X-ray photoelectron spectroscopy

**Figure 6.**
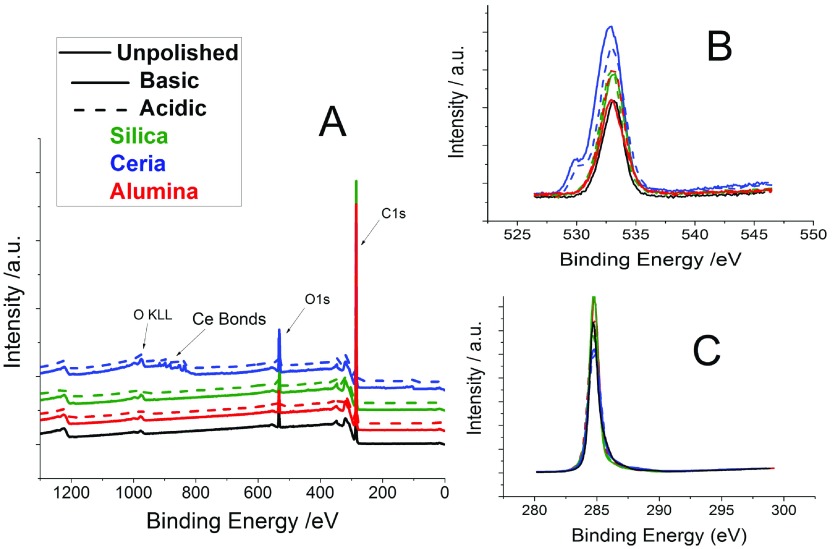
XPS data of the six polished films and an as-grown film. Panel (A) shows a sweep of the binding energy from 0 to 1200, revealing, on a broad spectrum, what is present on the surface of the diamond films. Panel (B) shows the O1s peaks and Panel (C) the C1s peaks.

XPS was taken of the six polished films and an as-grown film in order to compare the surface chemistry before and after polishing, the aim being to observe if the components from the slurry were bonding to the surface to facilitate CMP. The dust particles previously noticed by the AFM are highly unlikely to be the components of the slurries (ceria, silica or alumina) so there was no concern regarding contamination of the XPS data, the results of which are displayed in Figure 6.

Panel (A) shows that the only NCD thin films whose surface chemistry significantly changed were those polished by the ceria particle slurries. In the case of these two films, ceria particles remained bonded to the surface, detected around 900 eV, even after cleaning using the standard SC-1 process. There was a very weak 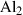 signal on the film that was polished using the basic alumina slurry but not for the acidic alumina. It is possible that the cleaning removed any trace of the Al and Si that was previously bonded to the surface.

Panel (B) depicts only the O1s signal of the surface; a sweep of binding energy from 520 up to 550 eV. The major peak at 532 eV corresponds to the C–O bond. A second peak can be observed at 530 eV corresponding to the Ce–O bond. Panel (B) shows that aside from ceria there is no other change to the type of oxygen bonds present on the surface. Panel (C) shows only the C1s signal with a sweep from 280 to 300 eV. Only the C–C bond is present with no other change observed before and after polishing.

Table 2 shows the O1s/C1s ratio for the unpolished film and the six polished films. Comparing these values shows how the oxygen environment relative to the carbon environment has changed after polishing. For all the films polished, there was an increase in the oxygen environment on the surface – implying the chemical nature of the technique. There was some variation between the oxidation levels. Those films polished by ceria show the highest increase to its oxygen environment. This is not surprising considering the ceria particles are still connected to the surface as well. The film polished with basic alumina shows very little oxygen increase.

**Table 2.** The O1s/C1s ratios for the films before and after polishing. The oxygen environment is seen to increase after polishing for all the films. For all the films polished, there was an increase in the oxygen environment on the surface – implying the chemical nature of the technique. There was some variation between the oxidation levels.

Polishing slurry	O1s/C1s ratio
None	0.1613
Basic alumina	0.1680
Acidic alumina	0.2022
Basic silica	0.2033
Acidic silica	0.2247
Acidic ceria	0.3123
Basic ceria	0.3567

### 3.3. Discussion

Thomas et al. proposed that the CMP of NCD thin films followed a mechanism similar to the CMP of silicon dioxide [17]. In this more traditional polishing process, the hydroxide ions within the polishing fluid react with the surface siloxane (Si–O–Si) bonds, creating a silanol based passivation layer (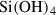) [26,27]. Silica particles within the polishing fluid will then attach themselves to the hydrated groups of the passivation layer. The polishing pad then introduces a shearing force on the said silica particles. If the energy from this shearing action is larger than the binding energy, the molecule will be removed, resulting in polishing of the surface. Thus, relating this process to diamond, a passivation layer would be formed by the increase of carbonyl and hydroxyl groups on the surface allowing the polishing slurry particles to bond to its surface.

Table 2 of this study shows that there is an increase in the oxygen content on the surface of all the diamond films, this is regardless of whether an acidic or basic slurry was used. In the case of ceria which remains on the surface, there is the greatest increase in oxygen content. The increase in oxygen suggests there is a reaction occurring on the surface of the NCD films which could be facilitating the polishing particles bonding to the surface, although there is no evidence for a passivation layer forming due to carbonyl and hydroxyl groups, otherwise there would have been a greater oxygen content on the surface of the films that were polished with the basic slurries. The basic slurries had a pH of only 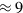, so it is possible a stronger basic solution could produce a passivation layer.

The polishing mechanism then requires, according to previous literature [17,26–28], the following relative differences in binding energies: C–C 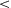 C–O 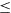 Ce–O/Si–O/Al–O. The current accepted values for the various bond strengths are shown in Table 3. Comparing the bond strength of the Al–O bond and the C–C bond (see Table 3) the C–C bond is clearly stronger and therefore Al–O should not be capable of polishing the diamond films if this were the polishing mechanism.

**Table 3.** The bond strength of the different bond types for each polishing particle [17,29].

Bond	Strength (kJ mol)
Ce–O	795
Al–O	511
Si–O	800
O–C	1077
C–C	610

This anomaly can be explained by recent results from Peguiron et al. [30]. The authors performed density functional theory calculations on the degradation of the diamond surface when in contact with silica and silicon, duplicating the model described by Thomas et al. [17]. They discovered that in a silicon particle bonded to diamond system, once a shearing force was applied this would always result in deformation of the silicon. However in the silica bonded to diamond system, once a shearing force was applied C–C bond breakage was shown, although this was not with great regularity. They determined that this is the result of activation (weakening) of C–C bonds between the terminating zigzag carbon chains and the underlying diamond bulk atoms. This weakening is two-fold requiring first that a pilot atom such as Si, O or H bond to the surface. The results of this study show oxygen bonding to the surface. It is possible that if the silicon is bonding to the surface it is being removed after being cleaned by the standard SC-1 process and hydrogen is not detectable by XPS. Then the weakening process requires that the pilot atom be attached to, or replaced by, a covalent and highly polar material, such as silica not silicon. This connection further weakens the C–C bond resulting in a breakage when a shearing force is applied. They concluded that polishing by this mechanism could also be achieved with alumina. This study has shown that it is possible to polish NCD films using alumina as predicted. This mechanism could provide an explanation for why ceria does not polish to the same rate as silica. Ceria contains both ionic and covalent bonding, with the ionic bond dominating [31]. This variation in bonding could also be the reason that the ceria remains bonded to the diamond film surface where the alternatives do not. It is possible that the covalent bonding, whilst not dominant, might be enough to provide the C -C bond weakening - since ceria still polishes at a similar (and in the case of the acidic ceria a greater) rate than the basic alumina slurry. The model proposed by Peguiron et al. [30] explains why alumina polishes, and possibly explains why ceria polishes, but it does not explain the variation between the alumina basic and acidic slurries. This variation can be explained if their particle size is considered in relation to the polishing rate, as Figure 5 clearly shows. This implies that the CMP of NCD thin films follows a contact-area mechanism where the roughness reduction is determined by the contact area of the particles with the sample, hence being inversely proportional to the particle size. This relationship has been observed in the CMP of other materials [32], although in cases apart from diamond the density and hardness of the polishing particles play a more significant role [32].

## 4. Conclusions

This study has shown that the CMP of NCD thin films is a chemical process that increases the O1s oxygen content on the surface of the films. Further changes have been proposed to the mechanism of the CMP of NCD thin films. Firstly it has been shown that CMP is not limited to slurries containing silica polishing particles. Three oxide polishing particles, ceria (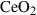), alumina (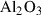) and silica (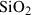), were tested under acidic and basic conditions. It was found that the acidic alumina slurry polished at a similar rate as the basic silica and acidic silica slurries, which agrees with separate theoretical predictions. However, the basic alumina polished at a significantly lower rate. This study shows that this is a result of the order of magnitude difference between the diameters of the polishing particles. It was found that of all the various properties of the slurries, including pH and composition, the particle size was a determining factor for the polishing rate, particles with a smaller diameter being capable of a greater RMS roughness reduction than those with a large diameter. This implies that the CMP of NCD thin films follows a contact-area mechanism where the rate of roughness reduction is determined by the contact area of the particles with the sample. Smaller particles are able to bond in greater numbers to the NCD thin film surface leading to more instances of C–C bond weakening and therefore successful polishing.
